# Protein knotting through concatenation significantly reduces folding stability

**DOI:** 10.1038/srep39357

**Published:** 2016-12-16

**Authors:** Shang-Te Danny Hsu

**Affiliations:** 1 Institute of Biological Chemistry, Academia Sinica, 128, Section 2, Academia Road, Taipei 11529, Taiwan

## Abstract

Concatenation by covalent linkage of two protomers of an intertwined all-helical HP0242 homodimer from *Helicobacter pylori* results in the first example of an engineered knotted protein. While concatenation does not affect the native structure according to X-ray crystallography, the folding kinetics is substantially slower compared to the parent homodimer. Using NMR hydrogen-deuterium exchange analysis, we showed here that concatenation destabilises significantly the knotted structure in solution, with some regions close to the covalent linkage being destabilised by as much as 5 kcal mol^−1^. Structural mapping of chemical shift perturbations induced by concatenation revealed a pattern that is similar to the effect induced by concentrated chaotrophic agent. Our results suggested that the design strategy of protein knotting by concatenation may be thermodynamically unfavourable due to covalent constrains imposed on the flexible fraying ends of the template structure, leading to rugged free energy landscape with increased propensity to form off-pathway folding intermediates.

Recent surveys of the Protein Data Bank have identified hundreds of toplogically knotted protein structures, accounting for nearly 1% of the total entries^1^,^2^. The knotted topologies range from the simplest trefoil (3_1_) knot, figure of eight (4_1_) knot, Gordian (5_2_) knot, to the most complex Stevedore’s (6_1_) knot^3^. Many knotted proteins exhibit well-characterised enzymatic functions involved in RNA methyl transfer, transcarbamylation, ubiquitin C-terminal hydrolysis, or dehalogenation^4^. Jackson and co-workers have elucidated extensively the folding pathways of two trefoil knotted bacterial methyl transferases, YibK from *Haemophilus influenzae* and YbeA from *Escherichia coli*^5^,^6^,^7^. Remarkably, YibK and YbeA remain knotted in the presence of concentrated chaotrophic agents, despite the lack of appreciable secondary and tertiary structures^8^,^9^,^10^. In fact, both proteins are random coil-like under chemically denatured states according to small angle X-ray scattering analyses^11^. Using a reconstituted *in vitro* transcription/translation system, it was recently demonstrated that YibK and YbeA can knot themselves spontaneously during *de novo* folding immediately upon completion of protein synthesis, while molecular crowding through encapsulation by the chaperonin system accelerates significantly the folding and knotting processes^12^. Since then, efforts have been made to characterize experimentally the folding dynamics and kinetics of knotted proteins with different degrees of complexities, including the smallest trefoil knot, MJ0366^13^, the most complex Stevedore’s knot, DehI^14^, and a family of Gordian knotted human ubiqtuitin C-terminal hydrolyases^15^,^16^,^17^. An emerging feature shared by all experimentally characterized knotted proteins is the presence of well-defined folding intermediate(s) along their folding pathways. This is consistent with theoretical predictions that knotting is the rate-limiting step along the multi-stage folding processes for most knotted proteins^18^,^19^,^20^,^21^,^22^,^23^.

In addition to naturally occurring knotted proteins, Yeates and co-workers reported the first example of a designed protein knot by concatenating an all-helical HP0242 from *Helicobacter pylori* that exists as an intertwined homodimer; the crystal structure of the concatenated variant is essentially identical to that of its parent with the flexible linker being too flexible to be defined in the crystal structure^24^,^25^. We have recently established the folding pathways of wild-type (wt) HP0242 and its concatenated form through multi-parametric spectroscopic analyses of the folding equilibria and kinetics induced by chemical denaturation^26^. HP0242 variants exhibit complex folding pathways with highly populated off-pathway folding intermediates that require back-tracking to attain native states, in line with theoretical predications^19^,^27^. To further examine in detail the folding of HP0242 variants and the impact of concatenation, we report here detailed folding analyses of HP0242 variants at atomic resolution by solution state NMR spectroscopy. Our results indicated that concatenation significantly destabilises the native structure of HP0242 to the extent that it resembles the global unfolding effect induced by concentrated chaotrophic agent, suggesting that knotting by concatenation may be thermodynamically unfavourable.

## Results

**Figure 1:**
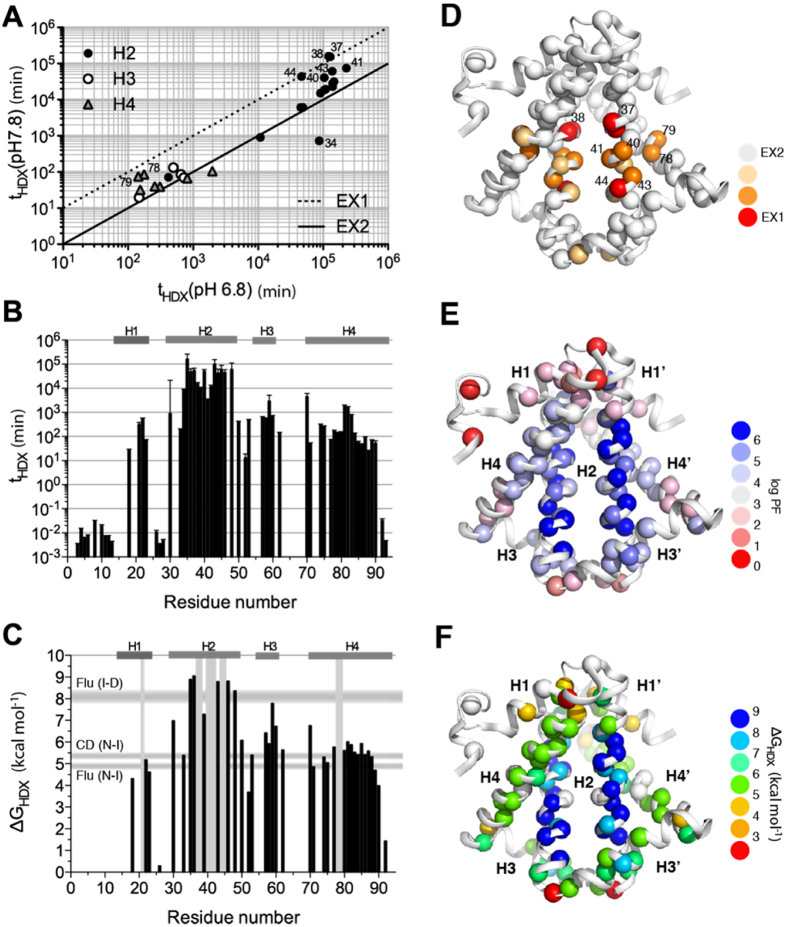
NMR HDX analysis of HP0242. (**A**) Comparison of the HDX time constants of HP0242 obtained at pH 6.8 and pH 7.8. The solid and dashed lines correspond to the expected correlations of the times constants under the EX2 and EX1 conditions. The residues in helices 2 (H2), 3 (H3) and 4 (H4) are shown in filled circles, open circle and grey triangle, respectively. Those that deviate significantly from the EX2 condition are labelled with the residue numbers. (**B**) HDX time constants as a function of residue number. (**C**) ΔG_HDX_ as a function of residue number. The locations of the EX1 residues are indicated by vertical grey bars. The ranges of free energies of unfolding for native-to-intermediate (NI) and intermediate-to-denatured (ID) derived from far-UV CD and intrinsic fluorescence are shown as horizontal grey bars as indicated on the left. (**D–F**) Structural mappings of the individual NMR HDX-derived parameters as shown in panels (A–C). The amide nitrogen atoms are shown in spheres with colour coding as indicated on the right hand side.

We have previously reported the backbone NMR assignments of wt HP0242 and the secondary structures in solution state are consistent with the crystal structure^28^. {^1^H}-^15^N heteronuclear nuclear Overhauser effect (hetNOE) of HP0242 confirmed that all the four helices are highly ordered on the timescale of ps-ns (Figure S1). Given that the unfolding kinetics of HP0242 variants under native conditions are on the timescale of hours to days, we opted to examine the slow folding dynamics of HP0242 by native NMR hydrogen-deuterium exchange (HDX)^16^,^17^,^29^ for slow-exchanging amide groups and phase-modulated clean chemical exchange (CLEANEX-PM)^30^ for fast exchanging amide groups, mostly the N-terminal region. The time constants of residue-specific amide HDX at pH 6.8 spanned from 10^2^ to 10^6^ minutes with those corresponding to the second helix (H2) being the slowest (Fig. 1). We repeated the same NMR HDX analysis at pH 7.8 to examine the pH-dependency of backbone amide HDX. While the HDX rates of most of the residues were proportional to catalyst concentration, *i.e.*, [OH^−^], indicating that the HDX process is under thermodynamic equilibrium, also known as the EX2 regime^31^, a few residues showed pH independent HDX rates, indicating that the HDX of these residues are under kinetic control of the opening rates of the corresponding hydrogen bonds, also known as the EX1 regime. For the EX2 residues, we calculated the protection factor (PF), and derived the corresponding free energy of unfolding, ΔG_HDX_, for individual backbone amide-mediated hydrogen bonds (see Methods). The results showed that H2, which is stabilised by leucine-zipper hydrophobic interactions has the higher free energy of unfolding up to 9 kcal mol^−1^, which is close to the bulk number corresponding to the transient between intermediate and denatured (I-D) states of HP0242 derived from intrinsic fluorescence^26^. The average free energy of unfolding of H1 and H4 is closer to the values corresponding to the native to intermediate (N-I) states of HP0242 derived from intrinsic fluorescence and far-UV circular dichroism spectroscopy. Collectively, these data reaffirmed the sequential unfolding pathway established by bulk spectroscopic measurements in which the peripheral helices, namely H1, H3 and H3 unfold first followed by unfolding of the long H2 that is stabilised by leucine zipper-based homodimerisation^26^.

**Figure 2:**
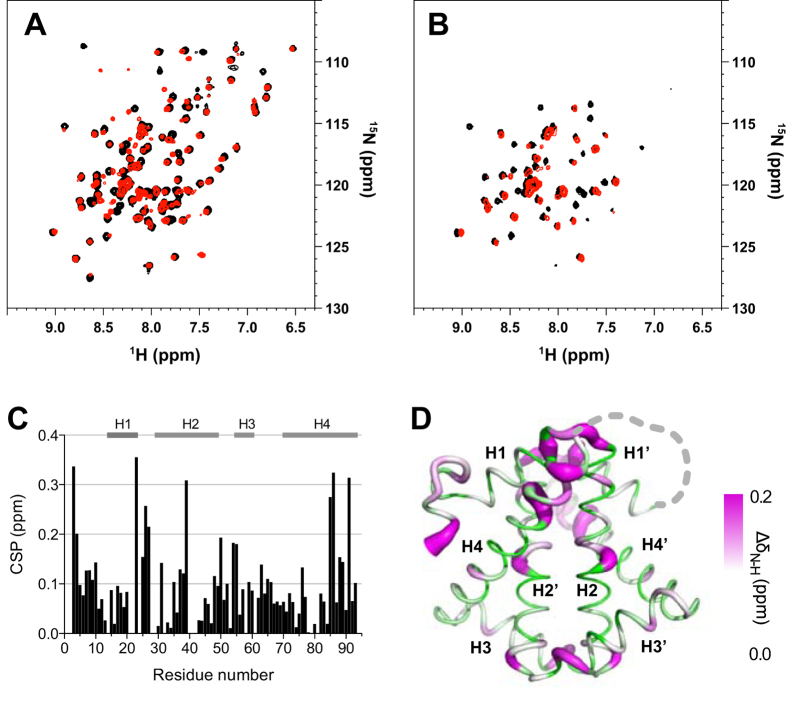
Impact of concatenation on HP0242. Overlay of ^15^N-^1^H correlation spectra of wt (black) and concatenated HP0242 (red) before (**A**) and after 10 min of HDX (**B**). (**C**) Weighted chemical shift perturbation (CSP) resulting from concatenation as a function of residue number. CSP is defined as Δδ_N-H_ = [(Δδ_N_)^2^ + (Δδ_H_/6.5)^2^]^1/2^. (**D**) Structural mapping of the observed CSP onto the crystal structure of concatenated HP0242 (PDB entry: 3MLG) with the radius of the sausage representation being proportional to the size of CSP as indicated on the right hand side of the colour scale. The sites of concatenation are connected by dashed grey line.

While our earlier work indicated that concatenated HP0242 exhibits more complex folding pathways with slower folding rates, possibly due to the increased likelihood of off-pathway folding intermediate formation, the molecular details of the impact on structure and folding dynamics upon concatenation of HP0242 remains elusive. We compared the backbone amide ^15^N-^1^H correlations of wt HP0242 and its concatenated form (Fig. 2). While the overall appearance of the two spectra were comparable, significant chemical shift perturbations were observed in the loop connecting H1 and H2 as well as most of H3, in addition to the flanking ends, *i.e.*, the N- and C-termini as a result of concatenation. Furthermore, minor cross-peaks were observed in the ^15^N-^1^H correlation spectrum of the concatenated HP0242 due to the loss of internal symmetry. Remarkably, despite the spectral similarity between native and concatenated HP0242, the latter showed dramatic loss of NMR signals after a short period (<10 min) of HDX in contrast to a much larger number of the remaining correlations for wt HP0242 dimer, indicating that concatenation significantly destabilises the native structure of HP0242 despite the essentially identical structures resolved in crystalline state under cryogenic conditions.

**Figure 3:**
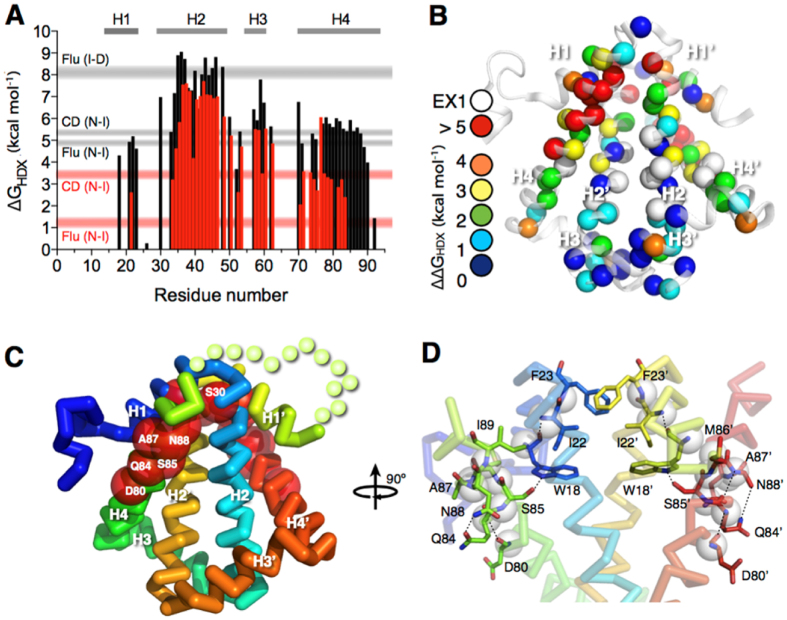
Concatenation destabilizes local structures of HP0242. (**A**) ΔG_HDX_ as a function of residue number. The results of wt and tandem HP0242 are shown in black and red bars, respectively. The ranges of free energies of unfolding for native-to-intermediate (NI) and intermediate-to-denatured (ID) derived from far-UV CD and intrinsic fluorescence are shown in horizontal grey (wt) and red (concatenated) bars as indicated on the left. (**B**) Structural mapping of the destabilization effect of concatenation. Individual backbone amide nitrogen atoms are shown in spheres and their respective ΔΔG_HDX_ = ΔG_HDX_(wt) − ΔG_HDX_(concatenated) values are colour-coded as indicated on the left. (**C**) Ribbon representation of concatenated HP0242 that is colour-ramped from blue to red for N- to C-termini, respectively. Residues that exhibit the largest destabilising effects are shown in red spheres and the identities are indicated in white letters. (**D**) Local structure of the destabilised region. Residues that are most destabilised are shown in stick representations and the inter-residue hydrogen bonds are shown in black dashed lines.

To further examine how concatenation impacts the folding of HP0242, we repeated the NMR HDX analysis for the concatenated form and found markedly reduced folding stabilities in H4 with its C-terminal half being so destabilised that no reliable HDX rates could be determined (Fig. 3). Likewise, most residues of H1 underwent rapid HDX that resulted in near-completely loss of protection against HDX. For H2 and H3, they were destabilised by as much as 2 kcal mol^−1^ across their whole sequences. Structural mapping of the residue-specific reduced free energy of unfolding showed that the impacts are localised near the concatenation site. Residues that are destabilised by more than 5 kcal mol^−1^ include S30, D80, Q84, S85, A87, N88 and I89. Other significantly destabilised residues (ΔΔG > 4 kcal mol^−1^) include W18, I22 and F23, all of which are located in close proximity to the only tryptophan residue of the protomer, W18. In addition to the backbone hydrogen bonds of the helical structure, the side-chain hydroxyl group of S85 is hydrogen bonded to the indole nitrogen of W18, whose intrinsic fluorescence serves as the structural probe in our earlier study^26^. It is likely that the introduction of covalent linkage between the C-terminus of one protomer to the N-terminus of the other through a short flexible linker imposes so much strain to the local structure around the C-terminal half of H4 that the corresponding hydrogen bond network is bulged from ideal geometry in solution.

**Figure 4:**
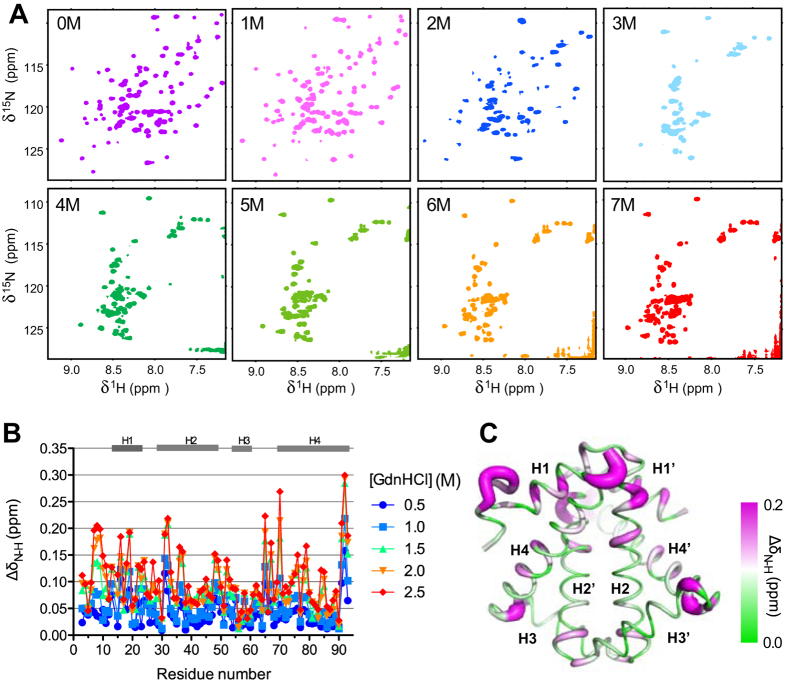
Chemical denaturation of HP0242 monitored by NMR. (**A**) A series of ^15^N-^1^H SOFAST-HMQC spectra were recorded for HP0242 in the presence of various amounts of GdnHCl as indicated in each panel. (**B**) Weighted chemical shift perturbation Δδ_N-H_ as a function of residue number, and denaturant concentration (from 0.5 to 2.5 M GdnHCl, with colour coding from blue to red as indicated on the right hand side). The residues that are in helical regions in the native state are indicated by grey bars on the top of panel B and numbered from H1 to H4 according to the reported the crystal structure of HP0242 (PDB entry: 2BO3). (**C**) Structural mapping of the weighted chemical shift perturbations of HP0242 in the presence of 2.5 M GdnHCl compared to those in native condition (red symbols in (**B**)). The size of the radius of the sausage representation corresponds to the magnitude of the chemical shift perturbation. It is also colour-ramped from green to magenta as indicated on the right hand side.

The large destabilising effect as a result concatenation prompted us to compare it with the destabilisation effect in response to chaotrophic agent-induced chemical denaturation. A series of ^15^N-^1^H correlation spectra of wt HP0242 were recorded in the presence of different concentrations of guanidine hydrochloride (GdnHCl) ranging from 0 to 7 M (Fig. 4). On increasing GdnHCl concentration, the intensities of well-dispersed ^15^N-^1^H correlations diminished progressively until they were too weak to be detected at 3 M GdnHCl. The chemical shift perturbations of individual ^15^N-^1^H correlations could be followed up to 2.5 M GdnHCl. Structural mapping of the observed chemical shift perturbations resemble remarkably with those observed in response to concatenation in that many of the highly perturbed residues are clustered around the junction between H1 and H4 where W18 is located. Several residues located at the fraying ends of the H3 and H2 also exhibited significant chemical shift perturbations. The loss of native ^15^N-^1^H correlation signals was accompanied by emergence of a group of poorly dispersed ^15^N-^1^H correlations, corresponding to unfolded population. Coexistence of native and chemically denatured ^15^N-^1^H correlations could be observed clearly at 2 M GdnHCl. Note however, that at near saturation denaturant concentration, *i.e.*, 7 M GdnHCl, the number of unfolded ^15^N-^1^H correlations was far smaller than the expected number for a fully disordered polypeptide chain of 92 residues in length, suggesting that a significant proportion of the chemically denatured HP0242 was in a molten globular state with abundant conformation exchange processes on the timescale of μs to ms, resulting in severe line broadening.

## Discussion

**Figure 5:**
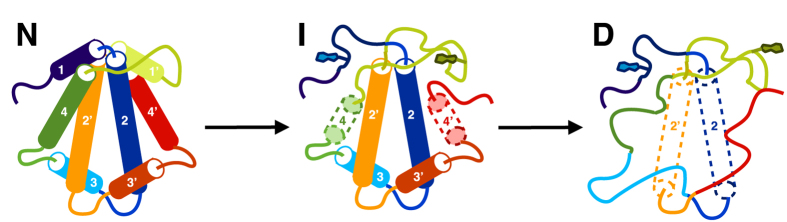
Proposed folding pathway of concatenated HP0242. The secondary structure elements are colour-ramped from blue to red from the N- to the C-termini with the helices numbered from one to four for the N-terminal half and those of the C-terminal half are numbered in the same way with additional apostrophes. The tryptophan side-chains are shown explicitly in the cartoon representation. In the intermediate (I) and denatured (D) states, the partially unfolded helices are shown in dashed cylinders.

In this work, we have compared the folding dynamics of wt and concatenated HP0242 using native NMR HDX analysis. Despite the essentially identical crystal structures of the two variants^24^, our results indicated that concatenation through a flexible linker between the two protomers results in substantial destabilisation across the entire structure with many residues showed reduced free energy of unfolding by more than 2 kcal mol^−1^ while the C-terminal half of H4 and most of H1, both of which are directly involved in concatenation, are destabilised by more than 4 kcal mol^−1^. The global destabilising effects led us to compare them with chaotropic agent-induced global destabilisation by NMR. The results revealed a high degree of similarity between the two types of destabilisation in terms of their spatial distributions on the structure of HP0242. Furthermore, significantly fewer than expected number of ^15^N-^1^H correlations in the presence of 7 M GdnHCl suggests the existence of residual structures, most likely populated within H2 due to its high stability derived from NMR HDX, that undergo helix-coil transitions on the μs to ms timescale, resulting in unfavourable line broadening beyond detection. Combining all the experimental evidence, we propose a linear folding pathway for concatenated HP0242 along which a partially unfolded folding intermediates become highly populated with the secondary structural elements around W18, namely H1, H4 and part of H2, being largely disordered. In the presence of highly concentrated GdnHCl (>6 M), subsequent unfolding of H3 and H2 takes place to form a molten globular denatured state (Fig. 5).

Protein repeats occur naturally through evolution^32^,^33^. Tremendous efforts have been made to engineer protein tandem repeats based on naturally occurring modules^34^,^35^,^36^,^37^,^38^,^39^,^40^ or *de novo* computational modules^41^,^42^. In most cases, the engineered protein tandem repeats display exceptionally high thermal and/or chemical stabilities compared to their ancestry building modules due to favourable enthalpic gains from inter-modular interactions and entropic stabilisation through covalent loop linkages^34^,^37^,^39^,^41^,^43^. A recent study by Tawfik and co-workers has nonetheless demonstrated through evolution traits that the stabilisation of the native states of β-propeller protein repeats is accompanied by parallel stabilisation of folding intermediates that are prone to misfold and aggregate^40^. Indeed, evolution tends to avoid high sequence similarity between neighbouring domains due to the higher propensity to misfold^44^,^45^. Theoretical and experimental analyses on the folding pathways of a series of circular permutations of β-trefoil interleukin-1β with different loop insertions suggested that these circular permutants tend to back-track on its folding landscape^46^, and that the destabilising effects can be attributed to geometric frustration of functional loops linking the modular repeats within the β-trefoil topology^47^. Collectively, these findings are in line with the fact that HP0242 exhibits complex folding pathways with high tendency to form off-pathway misfolded intermediates; the concatenated HP0242 exhibits significantly more populated folding intermediate than the intertwined symmetric dimer^26^. Our current findings highlighted the extra layer of complexity, in other words, energetic costs, involved in attaining the knotted topology of the concatenated form of HP0242. While knotting may be thermodynamically unfavourable, emerging evidence has revealed the functional importance of protein knots^48^ thus justifying their preservation throughout evolution.

## Methods

### Recombinant protein preparation

Uniformly ^15^N-labelled wt and concatenated HP0242 were over-expressed and purified according to the previously described protocol^26^,^28^. Unless otherwise specified, the NMR samples were buffered in 10 mM phosphate (pH 6.8) containing 10% D_2_O (v/v) and 0.02% NaN_3_.

### NMR spectroscopy

All NMR data were collected at 298 K using an AVANCE 800 (18.7 T), an AVANCE III 600 (14.0 T) or an AVANCE 500 (11.7 T) NMR spectrometer (Bruker Biospin, Germany). The latter two are equipped with a cryogen-cooled probe head. Unless otherwise specified, 5 mm quartz NMR tubes were used for data collection. The resulting datasets were processed by NMRPipe^49^ and analysed by Sparky^50^. {^1^H}-^15^N hetNOE was recorded at 14.0 T using parameters as described previously^51^.

### Chemical denaturation monitored by NMR spectroscopy

Aliquots of 0.1 mM ^15^N-labelled wt HP0242 were incubated in the presence of 0, 0.5, 1, 1.5, 2, 2.5, 3, 4, 5, 6 and 7 M GdnHCl overnight before NMR measurements. To minimize the interference from high salt contents during NMR measurements, 3 mm MATCH NMR tubes were used. ^15^N-^1^H SOFAST-HMQC^52^ spectra were recorded at 18.7 T (800 MHz proton Larmor frequency) using a room temperature probe. The chemical shifts of individual native backbone amide ^15^N-^1^H correlations were followed as a function of GdnHCl concentration up to 2.5 M at which point most of the native ^15^N-^1^H correlations are broadened beyond detection.

### NMR hydrogen exchange (HX)

The rates at which the backbone amide protons exchange with bulk solvent are determined HDX^29^,^31^ and CLEANEX-PM^30^. NMR HDX of wt and tandem HP0242 was carried out using the previously described protocol. Briefly, aliquots of pH-adjusted protein solution were lyophilized overnight and equal amounts of 99% D_2_O were added to resuspend the sample powder immediately before the NMR HDX measurements. The NMR HDX data were collected at 14.0 T by recording a series of ^15^N-^1^H SOFAST-HMQC spectra over a period of 20 days^29^. For fast HX processes of wt HP0242, CLEANEX-PM was used to determine the HX rates in 90% H_2_O and 10% D_2_O (v/v) at pH 6.8. The pH values of the samples were confirmed after the NMR HDX measurements. For both HP0242 variants, two pH values (7.8 and 6.8) were used to ascertain whether the subjects of interest are in the EX1 or EX2 regime. The HDX rate constants (*k*_*ex*_) of individual residues were used to derive the protection factors (PFs) using the Excel spreadsheet with built-in parameters that are available from the Englander group (hx2.med.upenn.edu/download.html). PF is the ratio of the intrinsic HDX rate (*k*_*int*_) over the observed HDX rate (*k*_*ex*_), PF = *k*_*int*_/*k*_*ex*_. For the EX2 residues, the corresponding PFs are subsequently converted into the free energy of unfolding, ΔG_HDX_, where, ΔG_HDX_ = −RT ln(PF).

## Additional Information

**How to cite this article**: Hsu, S.-T. D. Protein knotting through concatenation significantly reduces folding stability. *Sci. Rep.* **6**, 39357; doi: 10.1038/srep39357 (2016).

**Publisher’s note:** Springer Nature remains neutral with regard to jurisdictional claims in published maps and institutional affiliations.

## Supplementary Material

Supplementary Information
