# Supplementary material for: Protein knotting through concatenation significantly reduces folding stability
Source: Sci Rep. 2016 Dec 16;6:39357. doi: 10.1038/srep39357 (PMC5159899; doi:10.1038/srep39357)
Supplement: Supplementary Information [file srep39357-s1.doc]

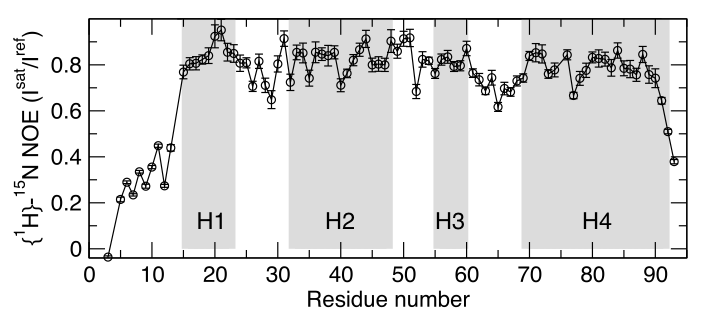


Figure S1. Backbone dynamics of HP0242 in solution. {^1^H}-^15^N heteronuclear NOE of HP0242 recorded at 14 T (^1^H Larmor frequency of 600MHz) and 298K.
